# Network pharmacology-based prediction and experimental validation of the anti-hyperuricemic effects of oolong tea polyphenols

**DOI:** 10.3389/fnut.2026.1845074

**Published:** 2026-08-11

**Authors:** Fenqi Lin, Duoer Lu, Jia Chen, Dongming Lu, Xiangjun Liu, Shiyu Lin, Langming Wu, Leisi Sun, Yueji Luo

**Affiliations:** 1 Changsha Medical University, Changsha, Hunan, China; 2 Hunan Provincial Key Laboratory of the Traditional Chinese Medicine Agricultural Biogenomics, Changsha Medical University, Changsha, Hunan, China

**Keywords:** dose-response, hyperuricemia, network pharmacology, oolong tea polyphenols, PI3K/Akt/mTOR signaling pathway, uric acid

## Abstract

**Objective:**

This study aimed to identify candidate therapeutic targets of oolong tea polyphenols (TP) against hyperuricemia (HUA) using network pharmacology and bioinformatics, and to validate the predicted molecular mechanism through *in vivo* experimentation.

**Methods:**

Drug and disease targets were retrieved from public databases, and overlapping targets were identified by Venn diagram analysis. Gene Ontology (GO) and Kyoto Encyclopedia of Genes and Genomes (KEGG) enrichment analyses were performed on the shared targets, and a protein-protein interaction (PPI) network was constructed to identify hub genes. For *in vivo* validation, an HUA mouse model was established by 15 days of oral potassium oxonate (PO) administration. Model mice then received TP by gavage at low (0.5 g⋅kg^−1^⋅d^−1^), medium (1 g⋅kg^−1^⋅d^−1^), or high (2 g⋅kg^−1^⋅d^−1^) doses for an additional 15 days. Serum biochemical markers, histopathological changes, and pathway-related protein expression were assessed by enzyme-linked immunosorbent assay (ELISA), hematoxylin and eosin (HE) staining, and western blot analysis, respectively.

**Results:**

Network pharmacology analysis identified 59 overlapping targets between TP and HUA; GO and KEGG enrichment analyses revealed that these targets were primarily associated with hormone metabolism and the PI3K-AKT signaling pathway. In the animal experiment, TP dose-dependently reduced serum uric acid (SUA) levels in hyperuricemic mice. At the molecular level, low and medium doses of TP suppressed phosphorylation of phosphatidylinositol 3-kinase (PI3K), protein kinase B (AKT), and mammalian target of rapamycin (mTOR), whereas the high dose paradoxically activated this pathway and concomitantly elevated interleukin-1β levels. These findings indicate that TP modulates uric acid metabolism through a non-monotonic, dose-dependent mechanism.

**Conclusion:**

By combining network pharmacology with animal experiments, this study identified the PI3K/AKT/mTOR signaling pathway as a likely mediator of the anti-hyperuricemic action of oolong tea polyphenols (TP). A medium dose of TP achieved the most balanced outcome, attenuating inflammation and preserving hepatic and renal architecture; the high dose, by contrast, paradoxically elevated interleukin-1β (IL-1β) and overactivated PI3K/AKT/mTOR signaling, underscoring the importance of dose calibration. These data suggest that a medium dose of TP may represent a feasible dietary strategy against hyperuricemia. Further work—including monomer identification, direct target validation, and clinical evaluation—is warranted to confirm and extend these preclinical findings.

## 1 Introduction

Hyperuricemia (HUA), a chronic metabolic disorder arising from dysregulated purine metabolism (1). With a prevalence reaching 14.0% among Chinese adults and notable sex-based disparities (2), HUA is classified by the 2018 European guidelines into HUA into asymptomatic and symptomatic stages (3), however, up to 50% of asymptomatic patients already exhibit urate deposition or bone erosion (4). Beyond gout, HUA correlates with heightened risks of cardiovascular events, cerebrovascular disease, and chronic kidney disease (5–7). Current pharmacotherapy remains limited, as first-line agents such as allopurinol carry risks of hypersensitivity and hepatorenal toxicity (8). Oolong tea polyphenols (TP), which are rich in catechins including epigallocatechin gallate (EGCG), epicatechin gallate (ECG), and epigallocatechin (EGC), have been reported to modulate renal urate transporter expression and reduce systemic inflammation (9). EGCG exerts metabolic benefits via PI3K/AKT-dependent mechanisms (10, 11), and dysregulation of the PI3K/AKT/mammalian target of rapamycin (mTOR) pathway has been implicated in HUA-associated renal and hepatic injury (12, 13). Despite these findings, it remains unknown whether a whole oolong tea polyphenol extract can target the PI3K/AKT/mTOR axis to simultaneously reduce uric acid levels and alleviate inflammation in HUA. We hypothesized that TP exert anti-hyperuricemic effects through dose-dependent modulation of the PI3K/AKT/mTOR pathway. This study integrates network pharmacology prediction with *in vivo* experimentation to identify candidate targets and validate the relevant molecular pathway (14).

## 2 Materials and methods

### 2.1 Prediction of potential targets and active components of oolong tea polyphenols

Based on published compositional data, seven bioactive compounds in oolong tea were selected for target prediction: epigallocatechin gallate (EGCG), gallocatechin gallate (GCG), epicatechin gallate (ECG), epigallocatechin (EGC), caffeine, epicatechin, and catechin. Molecular structures were retrieved from PubChem,^1^ and potential targets were predicted using PharmMapper^2^ and SwissTargetPrediction (15)^3^ (15). All predicted targets were then standardized to official gene nomenclature via UniProtKB.^4^

### 2.2 Acquisition of hyperuricemia-related targets

HUA-associated targets were retrieved by querying “hyperuricemia” across four databases: DrugBank,^5^ GeneCards,^6^ the Therapeutic Target Database (TTD),^7^ and PharmGKB.^8^ To ensure biological relevance, only targets with a relevance score ≥ 15 and a gene-disease association (GDA) score ≥ 0.01 were retained for downstream analysis.

### 2.3 Data intersection and GO and KEGG enrichment analyses

Gene Ontology (GO) and Kyoto Encyclopedia of Genes and Genomes (KEGG) pathway enrichment analyses were conducted using the clusterProfiler package in R, with an adjusted *p*-value threshold of 0.05 to define statistical significance. Enrichment results were visualized with the ggplot2 package. GO analysis encompassed all three ontology domains, namely cellular component (CC), molecular function (MF), and biological process (BP), thereby providing comprehensive functional annotation of the candidate targets.

### 2.4 Construction of the protein–protein interaction network

A protein-protein interaction (PPI) network was constructed using the STRING database^9^ (16). The 59 overlapping targets were submitted via the “multiple proteins” input mode, restricting the query to Homo sapiens. Interactions with combined confidence scores ≥ 0.7 were retained (highest ≥ 0.9; high ≥ 0.7; medium 0.4–0.7; low < 0.4) (17). From the resulting network, the largest connected component was isolated and subsequently imported into Cytoscape (17) for hub gene identification and topological analysis.

### 2.5 Reagents and instruments

#### 2.5.1 Experimental animals

Sixty male specific pathogen-free (SPF)-grade Kunming (KM) mice (body weight 18–20 g) were obtained from Changsha Tianqin Biotechnology Co., Ltd. (license No. SCXK (Xiang) 2022-0011). Mice were maintained in the animal facility of Changsha Medical University under controlled environmental conditions (approximately 25°C; 50–70% relative humidity) with *ad libitum* access to standard chow and water, and were acclimatized for 7 days prior to experimentation. All procedures were performed in accordance with the Animal Research: Reporting of *in vivo* Experiments (ARRIVE) guidelines and were approved by the Ethics Committee of Changsha Medical University (Approval No. D2023006).

#### 2.5.2 Materials

Oolong tea polyphenols (purity ≥ 98%; Shanghai Aladdin Biochemical Technology Co., Ltd.) were dissolved in 0.5% carboxymethyl cellulose sodium (CMC-Na) solution immediately before intragastric administration. The specific composition of the commercial extract was not independently characterized in the present study; this limitation was addressed in the Discussion.

#### 2.5.3 Reagents

Potassium oxonate (PO; purity ≥ 98%) and allopurinol tablets were used as the model-inducing agent and positive control drug, respectively. Assay kits for uric acid, blood urea nitrogen (BUN) were obtained from Beijing Solarbio Science and Technology Co., Ltd. Enzyme-linked immunosorbent assay (ELISA) kits for tumor necrosis factor-α (TNF-α), cyclooxygenase-2 (COX-2), interleukin-1β (IL-1β) were sourced from Shanghai Jianglai Biotechnology Co., Ltd. Sodium carboxymethyl cellulose (CMC-Na) was obtained from Dalian Meilun Biotechnology Co., Ltd. Rabbit polyclonal antibodies against AKT, p-AKT, PI3K, p-PI3K, mTOR, and p-mTOR, together with horseradish peroxidase-conjugated goat anti-rabbit IgG secondary antibodies, skim milk powder, broad-spectrum prestained protein marker, bicinchoninic acid (BCA) protein assay kit, radioimmunoprecipitation assay (RIPA) lysis buffer, protease inhibitor cocktail, phosphatase inhibitor cocktail, and enhanced chemiluminescence reagent, were purchased from Wuhan Servicebio Technology Co., Ltd.

#### 2.5.4 Instruments

Major instruments included: an Eppendorf Centrifuge 5810R refrigerated centrifuge, FA1004 electronic analytical balance, electric thermostatic water bath, freeze dryer, Fisher Scientific PowerGen125 high-speed homogenizer, 201-II rotary evaporator, Motic BA210T biological microscope, BMJ-A tissue embedding machine, YD-315 rotary microtome, DHP-500 constant-temperature incubator, L530 automatic balancing centrifuge, SHB-3 circulating water vacuum pump, UV-1100 ultraviolet-visible spectrophotometer, DL-1 universal electric furnace, 78-1 magnetic heating stirrer, HH-S digital thermostatic water bath, FW80 high-speed universal grinder, a mini vertical electrophoresis apparatus, and a semi-dry transfer system.

### 2.6 Model establishment and group administration

After 7 days of acclimatization, KM mice were randomly allocated to six groups (*n* = 10 per group): normal control, model, allopurinol, and low-, medium-, and high-dose TP. During the model induction phase (days 1–15), model group mice received PO (300 mg/kg) suspended in 0.50% CMC-Na solution by oral gavage at a volume of 0.1 mL per 10 g body weight once daily; normal control mice received an equivalent volume of vehicle (0.50% CMC-Na) on the same schedule. On day 15, a subset of model group mice were anesthetized with 2% sodium pentobarbital at 1 h post-gavage, and blood was collected via retro-orbital enucleation, followed immediately by euthanasia via cervical dislocation. Serum was separated after standing at room temperature and assayed for SUA and BUN. Significantly elevated SUA levels in model mice relative to normal controls confirmed successful HUA induction. During the treatment phase (days 16–30), PO administration was continued in all model groups, and mice in the low-, medium-, and high-dose TP groups additionally received 0.5, 1, and 2 g⋅kg^−1^⋅d^−1^ TP, respectively. On day 30, at 1 h after the final administration, all remaining mice were anesthetized, and blood was collected via retro-orbital enucleation followed immediately by euthanasia via cervical dislocation. Serum was obtained by centrifugation at 3,000 r⋅min^−1^ for 15 min and stored at −20°C for subsequent biochemical analysis. Liver and kidney tissues were excised and fixed in 10% paraformaldehyde.

### 2.7 Determination of uric acid and blood urea nitrogen

Serum uric acid and BUN concentrations were determined using commercial assay kits in accordance with the manufacturers’ instructions.

### 2.8 ELISA detection of IL-1β, TNF-α, COX-2, and XOD in kidney tissue

IL-1β, TNF-α, COX-2, and XOD concentrations in renal tissue homogenates were quantified by ELISA following the manufacturers’ protocols.

### 2.9 Preparation of liver and kidney tissue sections

Paraffin-embedded tissue sections previously fixed in 10% paraformaldehyde were heated at 60°C for 1–2 h. Sections were deparaffinized through two 10-min xylene washes, then rehydrated by sequential immersion in graded ethanol (100, 95, 85, and 75%; 5 min each). After a 5-min rinse in distilled water, sections were stained with hematoxylin for 5–10 min, rinsed, and blued in phosphate-buffered saline. Eosin counterstaining was performed for 3–5 min, followed by dehydration through ascending ethanol concentrations (95–100%), two 10-min xylene clearings, neutral resin mounting, and light microscopic examination.

### 2.10 Renal tissue expression of PI3K/AKT/mTOR pathway proteins assessed by western blot

Total protein was extracted in RIPA lysis buffer supplemented with protease inhibitors; nuclear and cytoplasmic fractions were separated using a commercial extraction kit. Protein concentrations were determined by BCA assay, and equal amounts of denatured protein were resolved by sodium dodecyl sulfate-polyacrylamide gel electrophoresis (SDS-PAGE; 10% separating gel) before transfer onto polyvinylidene difluoride (PVDF) membranes. Membranes were blocked with 5% skim milk for 1 h at room temperature, then incubated overnight at 4°C with the appropriate primary antibodies. The following day, membranes were equilibrated to room temperature for 1 h, washed three times with Tris-buffered saline containing 0.1% Tween 20 (TBST), and probed with goat anti-rabbit IgG secondary antibody (1:3,000) for 1 h. Immunoreactive bands were visualized by enhanced chemiluminescence and captured with a digital imaging system. Densitometric analysis was performed using GeneTools software, and all experiments were conducted in triplicate.

### 2.11 Statistical analysis

All quantitative data are expressed as mean ± standard deviation (x̄ ± s). Statistical analyses were conducted in R (version 4.4.1) and GraphPad Prism (version 9.5). Intergroup differences were evaluated by one-way analysis of variance (ANOVA), followed by Dunnett’s *post hoc* test for pairwise comparisons of each treatment group against the normal control. Statistical significance was set at *P* < 0.05, with *P* < 0.01 denoting a highly significant difference.

## 3 Results

### 3.1 Screening of active components and targets

#### 3.1.1 Major components of oolong tea polyphenols and numbers of corresponding targets

Potential molecular targets of the seven oolong tea components were predicted using PharmMapper, SwissTargetPrediction, and TargetNet. After gene name standardization through UniProtKB, a total of 754 unique targets associated with oolong tea polyphenols were retained (Supplementary Table 1).

#### 3.1.2 Number of HUA-related targets

HUA-associated targets were retrieved from four public databases: DrugBank, GeneCards, the Therapeutic Target Database (TTD), and PharmGKB. Integration of results across databases and removal of duplicates yielded 1,266 non-redundant disease targets (Supplementary Table 2).

#### 3.1.3 Venn diagram of intersecting targets

**FIGURE 1:**
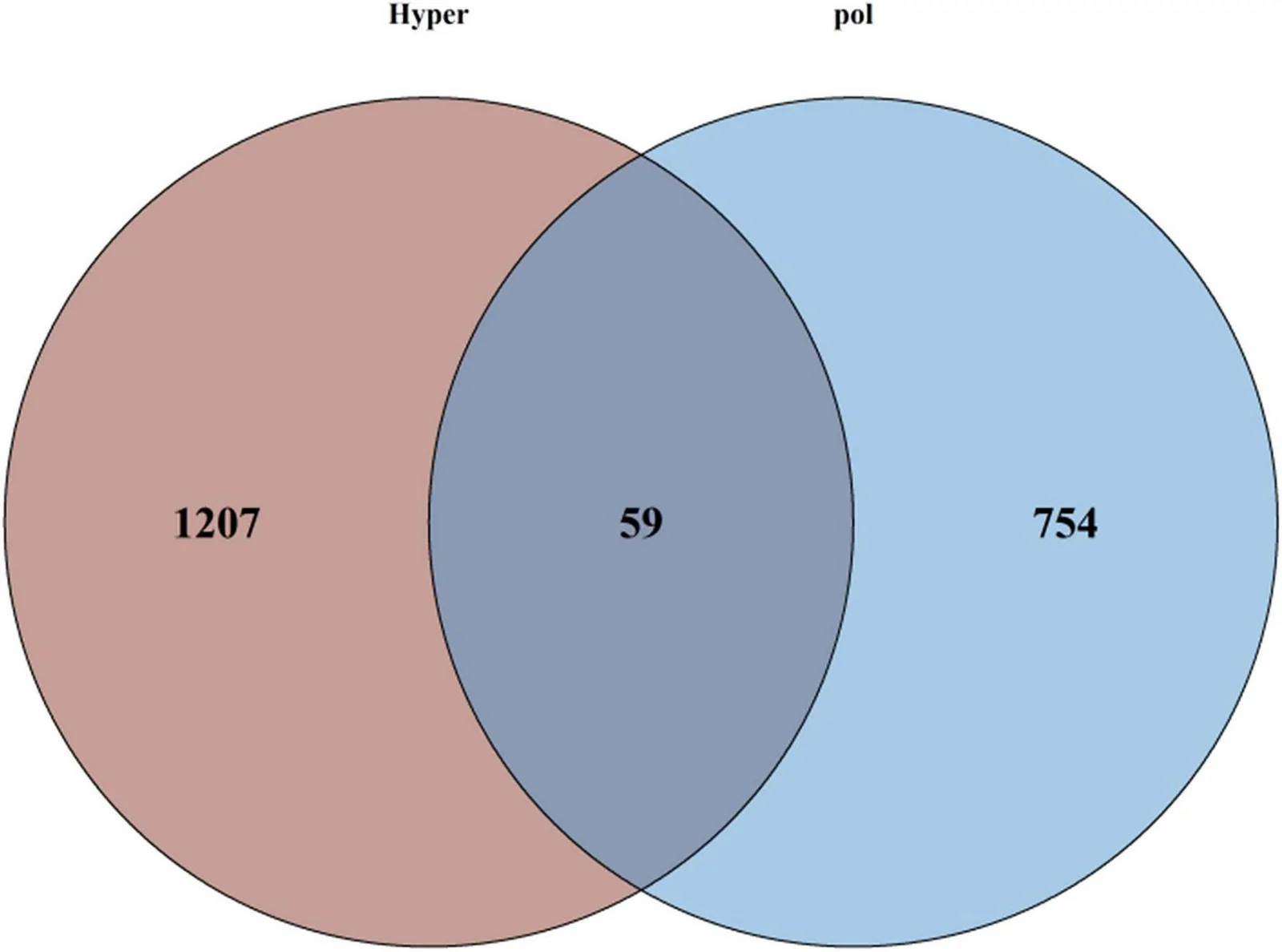
Venn diagram of oolong tea polyphenol targets and HUA-related targets.

Intersection of oolong tea polyphenol targets with HUA-associated targets yielded 59 overlapping genes as candidate therapeutic targets (18, 19) (Figure 1).

### 3.2 Enrichment analysis results

#### 3.2.1 Results of GO enrichment analysis

**FIGURE 2:**
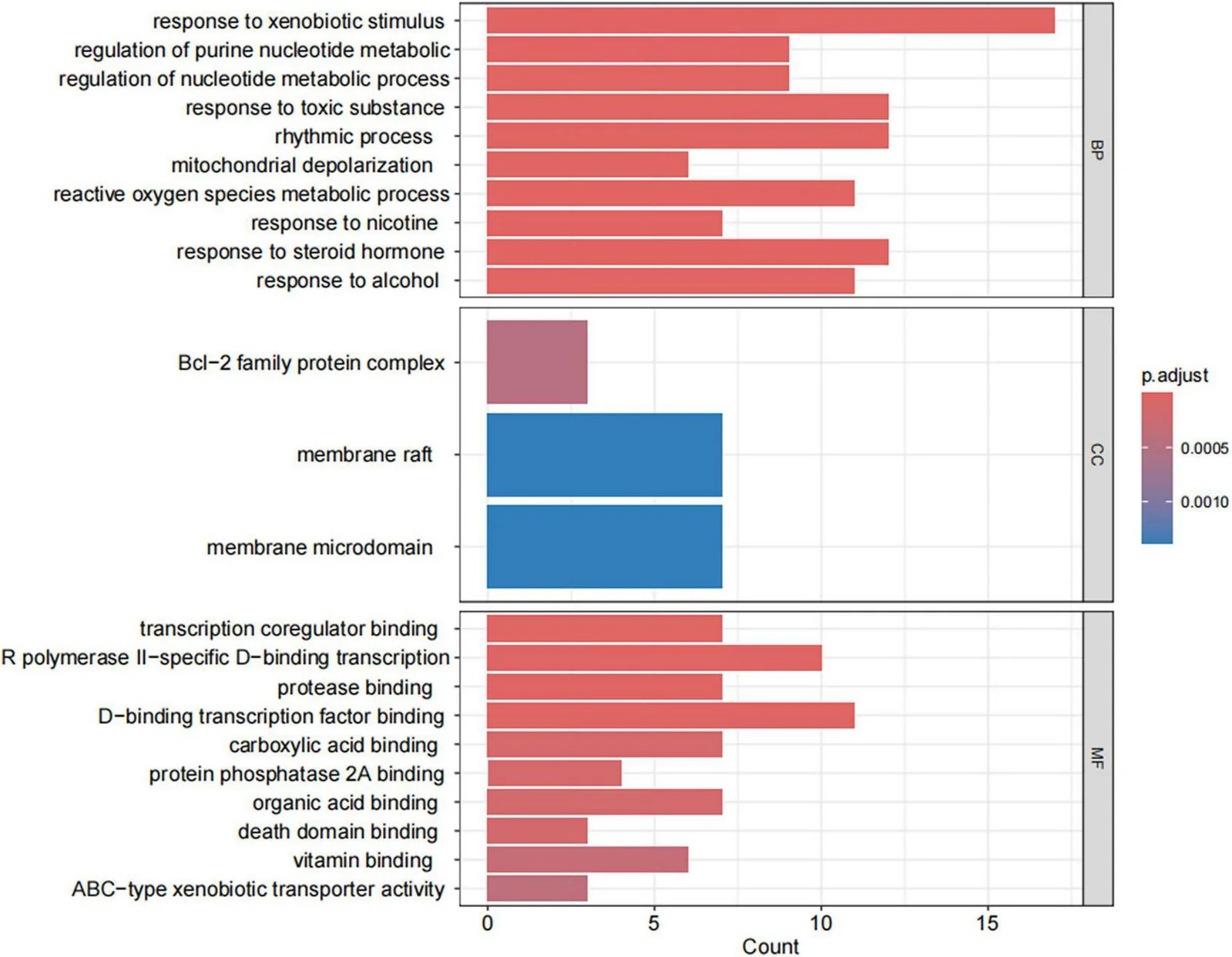
Results of GO enrichment analysis.

GO enrichment analysis of the 59 candidate targets returned 851 significantly enriched terms: 809 in biological process (BP), 39 in molecular function (MF), and 3 in cellular component (CC) (top-10 *p*-values all < 0.05). The most prominently enriched terms (Figure 2) encompassed xenobiotic stimulus response, nucleotide metabolic process regulation, toxic substance response, Bcl-2 family protein complexes, membrane rafts and microdomains, transcriptional coregulator binding, RNA polymerase II-specific DNA-binding transcription factor binding, and protease binding.

#### 3.2.2 Results of KEGG enrichment analysis

**FIGURE 3:**
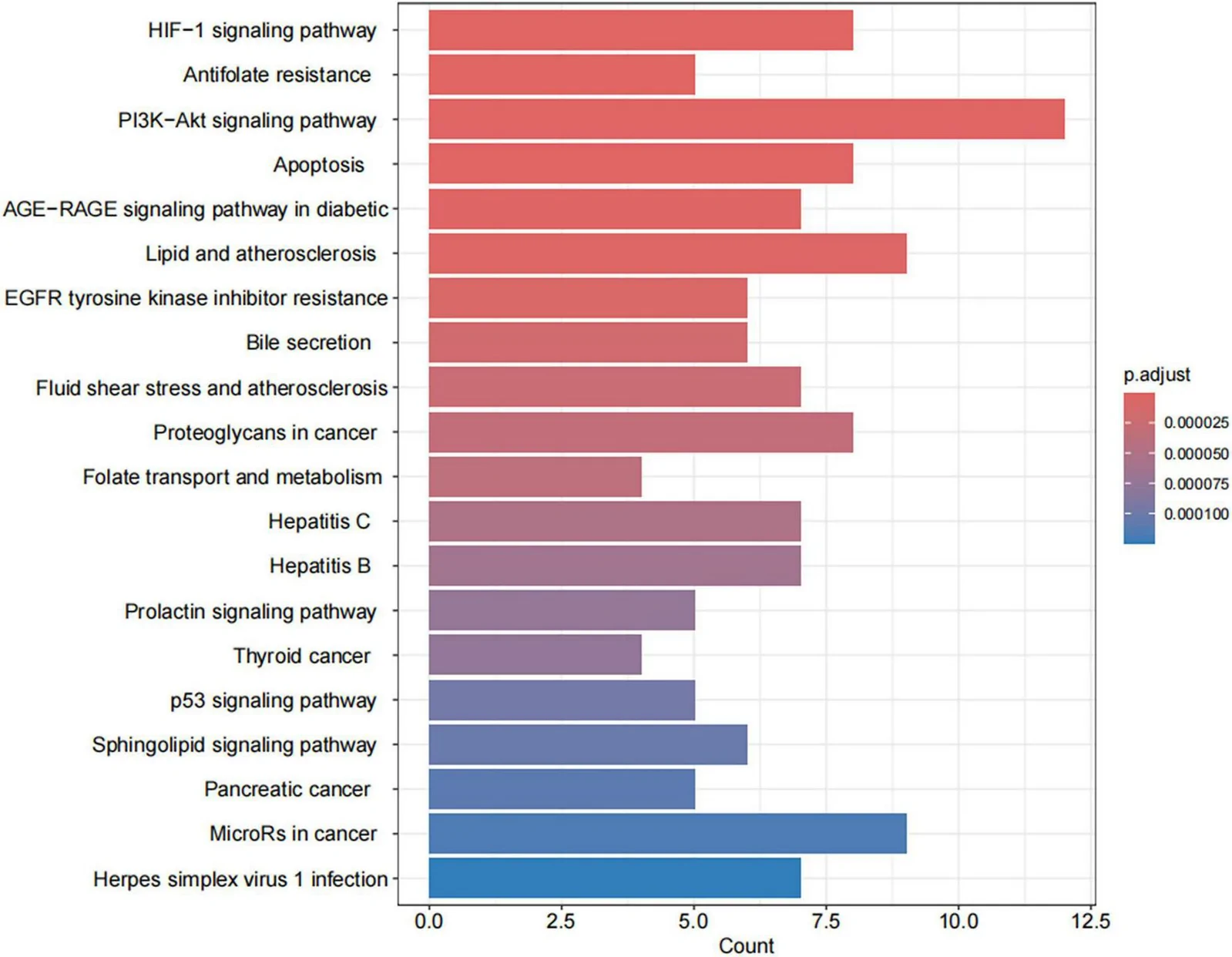
Results of KEGG enrichment analysis.

KEGG pathway enrichment analysis, performed in RStudio, covered five major pathway categories: metabolism, genetic information processing, environmental information processing, cellular processes, and organismal systems. Of the 63 significantly enriched pathways identified, HIF-1 signaling, PI3K-AKT signaling, AGE-RAGE signaling in diabetic complications, and apoptosis ranked among the most highly enriched (Figure 3).

### 3.3 Construction of the protein–protein interaction network

**FIGURE 4:**
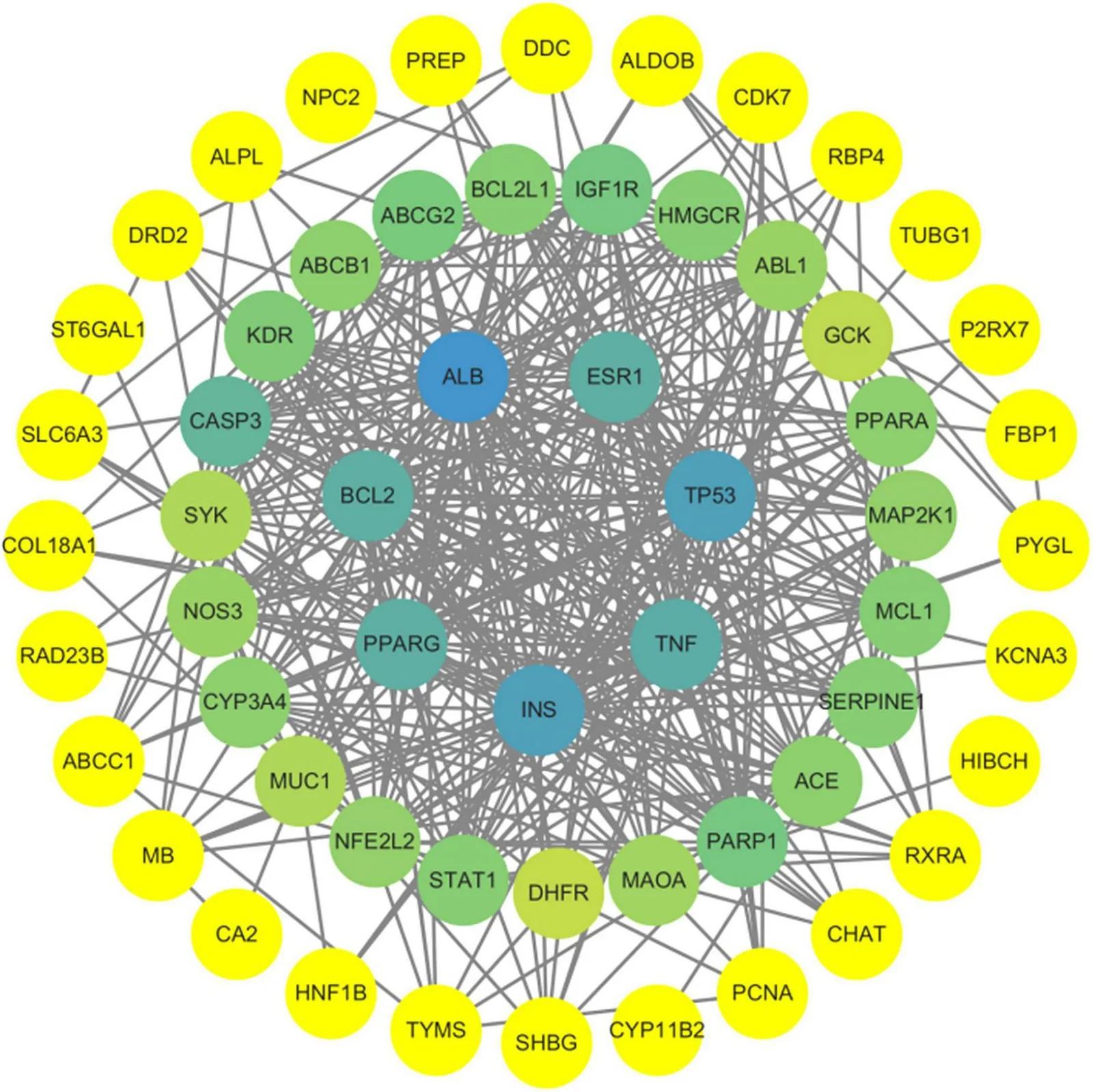
Protein–protein interaction network.

A protein-protein interaction (PPI) network was constructed to visualize interactions among the 59 candidate targets and to identify core hub genes. The resulting network encompassed 384 edges connecting 58 nodes (Figure 4); node color intensity scaled from light to dark in proportion to degree centrality. Hub genes were subsequently identified using the cytoHubba plugin in Cytoscape 3.10.3, which computed mean topological parameters for each node.

### 3.4 Effects of TP on serum uric acid and blood urea nitrogen levels in mice

To assess the urate-lowering efficacy of oolong tea polyphenols (TP), serum uric acid (SUA) and blood urea nitrogen (BUN) were measured. As shown in Table 1, SUA in the model group was markedly elevated relative to normal controls (*p* < 0.01), confirming successful induction of hyperuricemia. Compared with the model group, SUA was significantly reduced in all three TP-treated groups (all *p* < 0.05); the high-dose group showed the largest decrease, falling to 405 μmol/L. BUN was also markedly higher in the model group than in normal controls, indicating renal impairment, whereas the medium-dose, high-dose, and allopurinol groups returned to near-normal levels.

**TABLE 1:** Effects of TP on SUA and BUN levels in HUA mice.

Group	SUA (μmoL/L)	BUN (mmoL/L)
Normal	180.00 ± 36.59	5.16 ± 0.17
Model	600.49 ± 14.53	8.37 ± 0.23
Low	577.14 ± 12.51	6.05 ± 0.19
Medium	428.57 ± 11.35	5.576 ± 0.033
High	405.72 ± 1.50	5.324 ± 0.096
Allopurinol	216.96 ± 22.50	5.070 ± 0.036

### 3.5 Effects on hepatic and renal tissue morphology in HUA model mice

**FIGURE 5:**
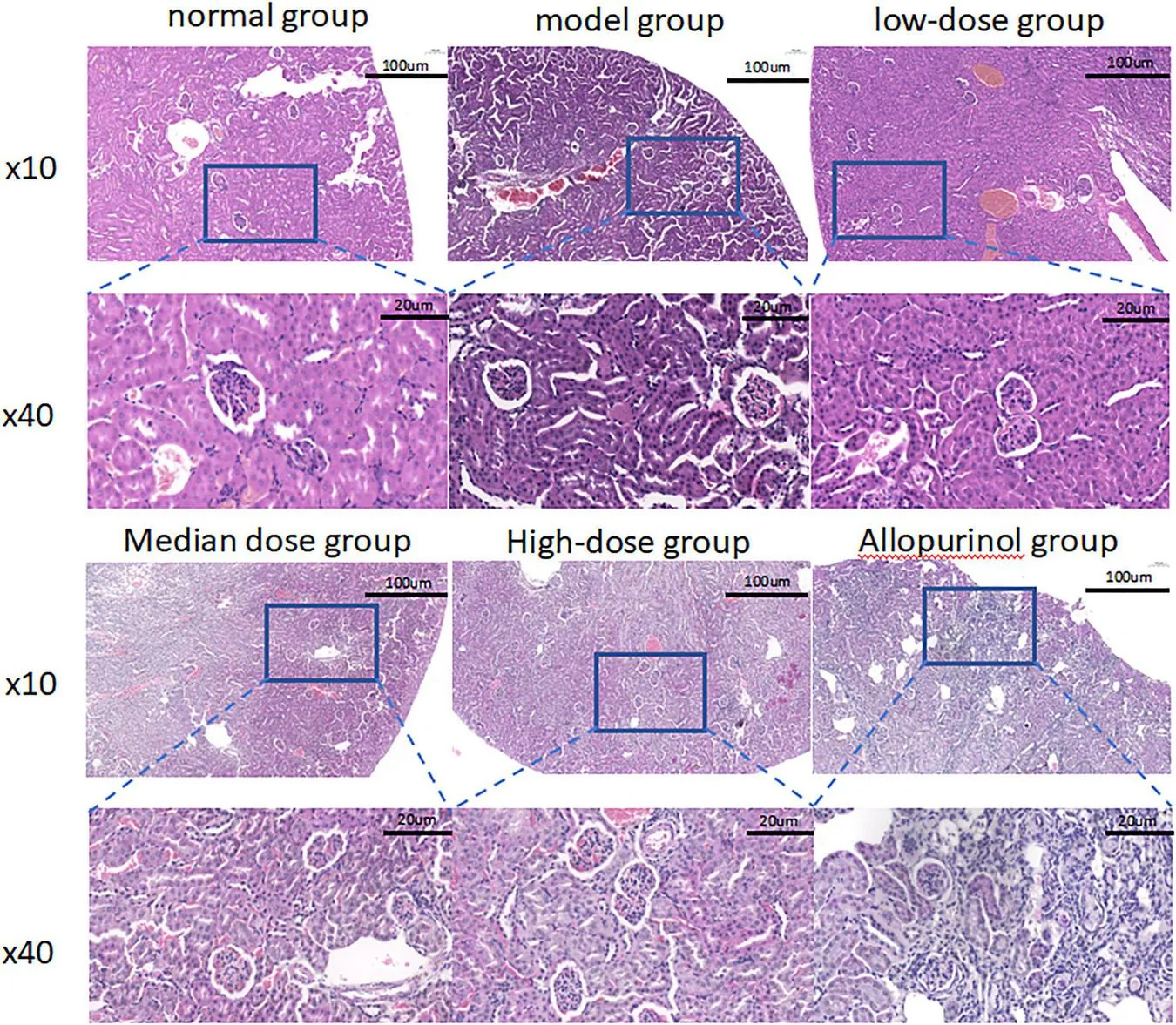
Renal tissue sections of HUA model mice.

As shown in Figure 5, glomeruli, renal tubules, and interstitium in the normal group exhibited intact structures without deposition or inflammation. In the model group, extensive tubular necrosis, massive urate crystal obstruction of tubular lumens, dense interstitial inflammation, and fibrosis. The low-dose TP treatment group exhibited pathological changes similar to those in the model group, with marked tubular necrosis and interstitial fibrosis persisting. In the medium-dose TP treatment group, moderate tubular degeneration and crystal deposition were observed, accompanied by moderate interstitial inflammation and fibrosis. In the high-dose TP treatment group, only mild protein deposition was observed in renal tubules, slight interstitial edema, and well-preserved glomerular architecture. In the allopurinol positive control group, mild degeneration of tubular epithelial cells with limited crystal deposition and mild interstitial inflammation and fibrosis.

**FIGURE 6:**
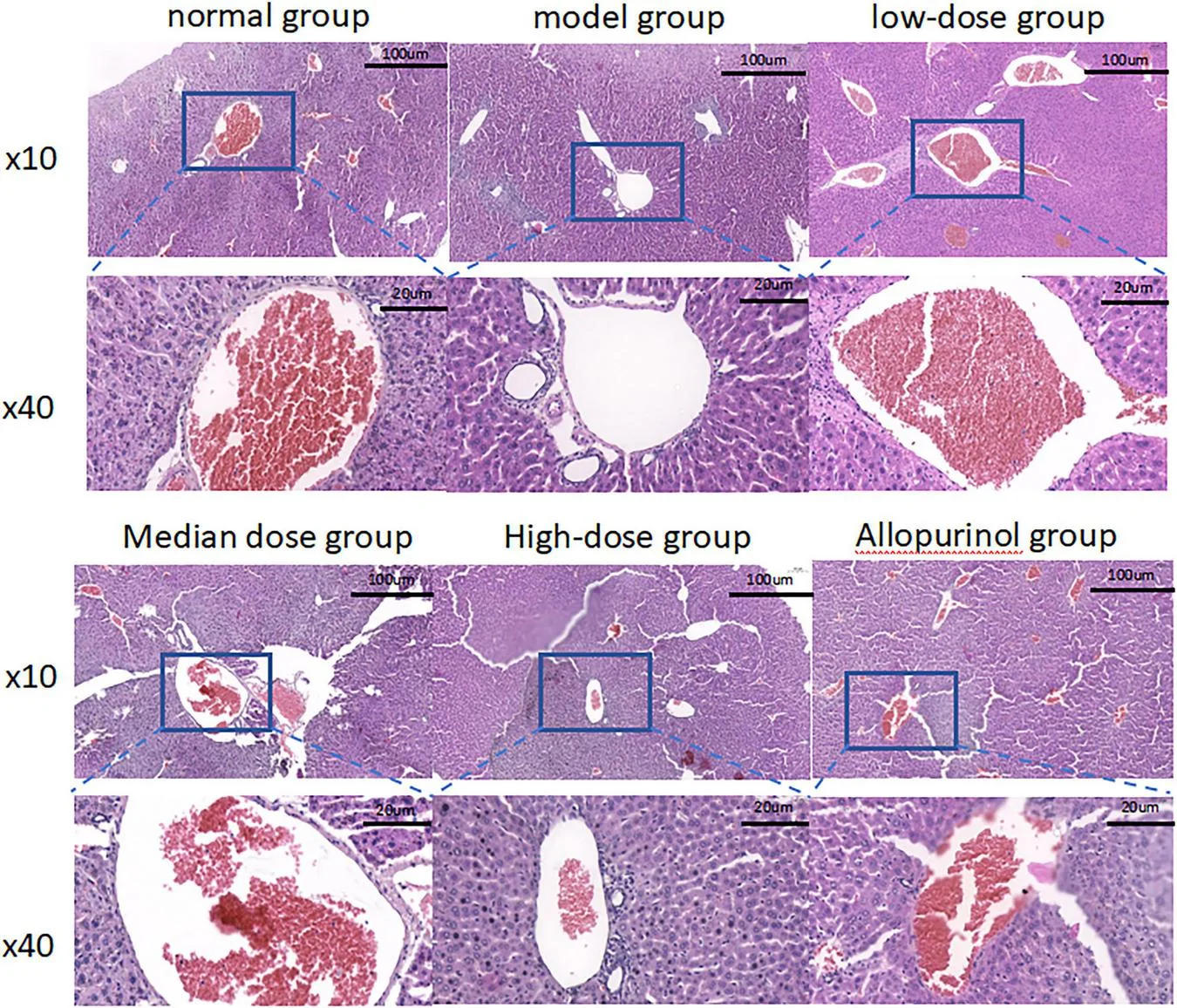
Hepatic tissue sections of HUA model mice.

Figure 6 demonstrates that hepatic tissue in the normal group maintained normal architecture, characterized by clear lobular structure, orderly hepatocyte arrangement, and no evidence of steatosis, inflammation, or fibrosis. In the model group, marked inflammatory infiltration, severe steatosis, and aggravated portal fibrosis with fibrous septa formation were observed. The low-dose TP group displayed only mild steatosis, with no inflammation or fibrosis and intact lobular structure. In the medium- and high-dose TP treatment groups, hepatic tissue architecture was essentially normal, with no evident steatosis, inflammation, or fibrosis, resembling the normal group. In the allopurinol positive control group, moderate steatosis, mild portal inflammation, and moderate fibrosis were observed.

### 3.6 Effects on renal inflammatory factor levels in HUA model mice

As shown in Table 2, renal IL-1β, TNF-α, COX-2, and XOD levels were all elevated in the model group relative to normal controls, consistent with hyperuricemia-induced renal inflammation and disturbed urate metabolism. All three doses of oolong tea polyphenols (TP) reduced these inflammatory mediators to varying extents, with the medium dose producing the most marked suppression of TNF-α, COX-2, and XOD. The high-dose effect on IL-1β was attenuated, with levels approaching those of the model group, suggesting that TP regulates renal inflammation in a non-monotonic, dose-dependent manner.

**TABLE 2:** Tea polyphenol modulation of inflammatory factor expression (IL-1β, TNF-α, COX, and XOD) in renal tissue of HUA model mice.

Group	IL-1β (ng/g)	TNF-α (ng/g)	COX-2 (ng/g)	XOD (U/g)
Normal	164.70 ± 6.90	783.13 ± 9.36	277.00 ± 5.91	1.83 ± 0.23
Model	191.24 ± 4.66	853.73 ± 9.66	308.46 ± 6.77	2.05 ± 0.29
Low	167.82 ± 7.92	668.07 ± 26.56	292.53 ± 7.05	0.92 ± 0.21
Medium	171.50 ± 5.37	655.94 ± 23.55	241.17 ± 10.29	0.59 ± 0.01
High	189.84 ± 3.80	697.44 ± 0.72	262.80 ± 10.53	0.80 ± 0.16
Allopurinol	141.67 ± 1.28	568.97 ± 28.00	207.77 ± 2.22	1.18 ± 0.20

### 3.7 Effects on serum inflammatory factor levels in HUA model mice

As shown in Table 3, serum IL-1β, TNF-α, COX-2, and XOD were all elevated in the model group relative to normal controls, consistent with a systemic inflammatory response to hyperuricemia. Medium-dose TP brought serum IL-1β and TNF-α back to near-normal levels, whereas TNF-α in the high-dose group remained comparable to that of the model group; changes in COX-2 and XOD were modest across all TP doses. Allopurinol produced the strongest reductions in TNF-α, COX-2, and XOD. Overall, the medium dose was most effective at lowering serum IL-1β and TNF-α, whereas the impact of TP on COX-2 and XOD remained limited across the dose range.

**TABLE 3:** Tea polyphenol modulation of serum inflammatory factor levels (IL-1β, TNF-α, COX, and XOD) in HUA model mice.

Group	IL-1β (ng/g)	TNF-α (ng/g)	COX-2 (ng/g)	XOD (U/g)
Normal	27.65 ± 6.63	95.12 ± 29.62	1.65 ± 0.42	0.33 ± 0.08
Model	48.84 ± 27.12	126.24 ± 42.19	2.84 ± 1.15	0.60 ± 0.10
Low	27.35 ± 10.79	121.54 ± 18.66	2.68 ± 1.98	0.55 ± 0.05
Medium	19.51 ± 0.69	95.05 ± 10.27	2.80 ± 0.61	0.54 ± 0.05
High	21.13 ± 2.74	126.87 ± 14.52	2.99 ± 0.97	0.52 ± 0.03
Allopurinol	18.12 ± 7.17	60.15 ± 16.37	1.61 ± 0.29	0.27 ± 0.27

### 3.8 Effects on the PI3K/AKT/mTOR signaling pathway in renal tissue of HUA model mice

**FIGURE 7:**
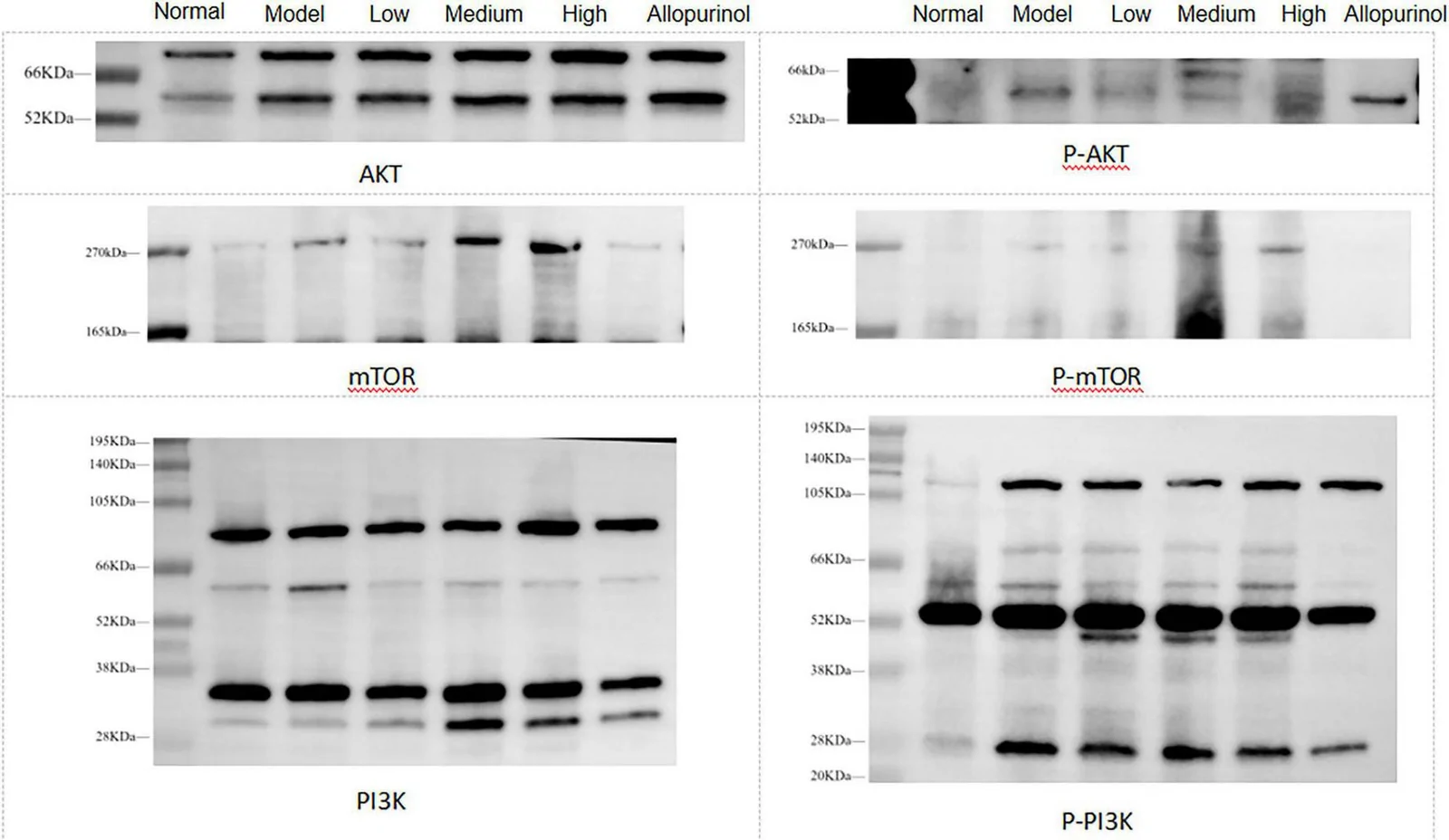
Effects of oolong tea polyphenols on PI3K/AKT/mTOR pathway protein expression in renal tissue of HUA model mice.

**FIGURE 8:**
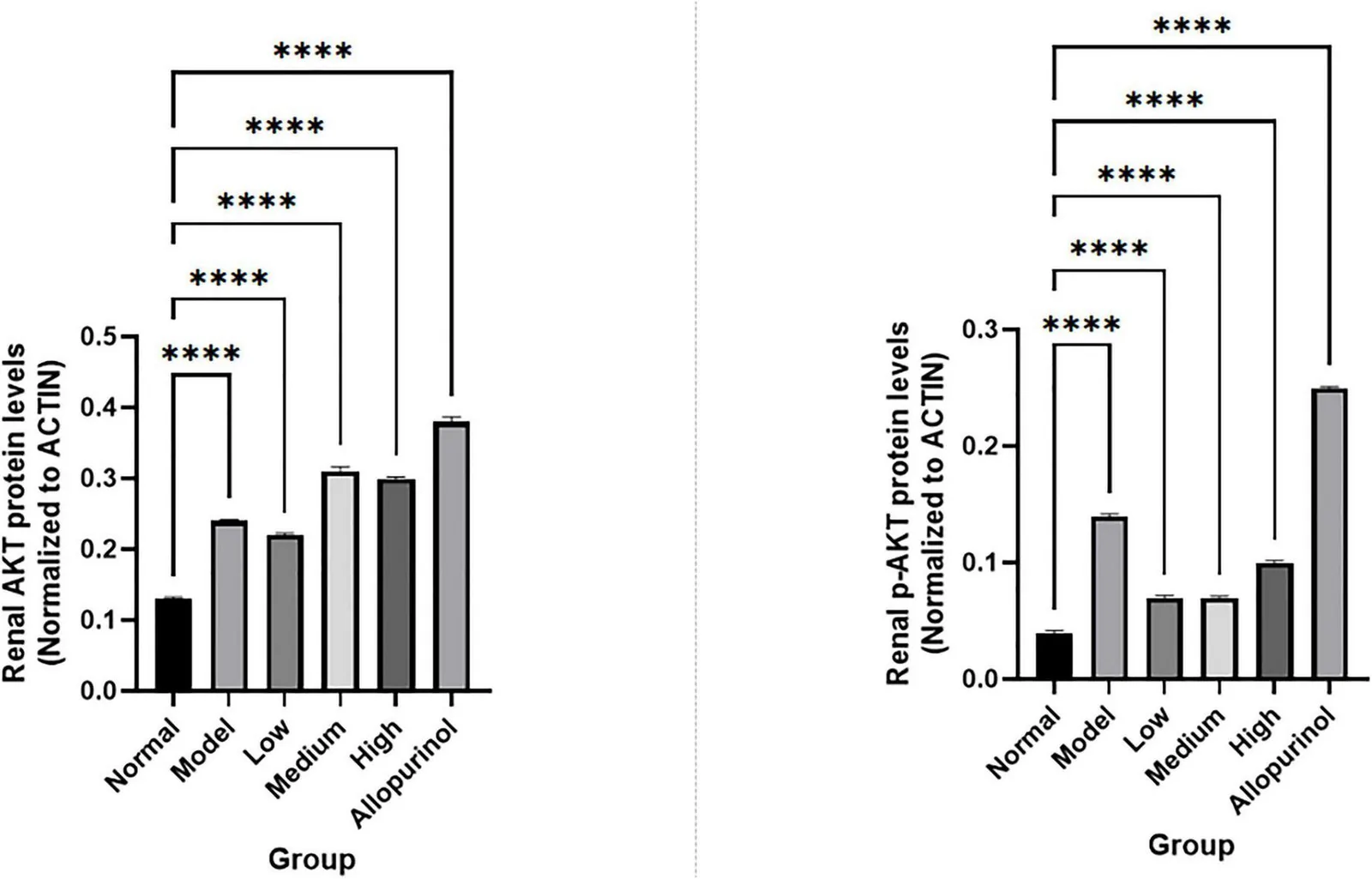
Effects of tea polyphenols on protein expression levels of AKT, p-AKT in HUA mice. ****Denotes a statistically significant difference at the *p* < 0.0001 level.

**FIGURE 9:**
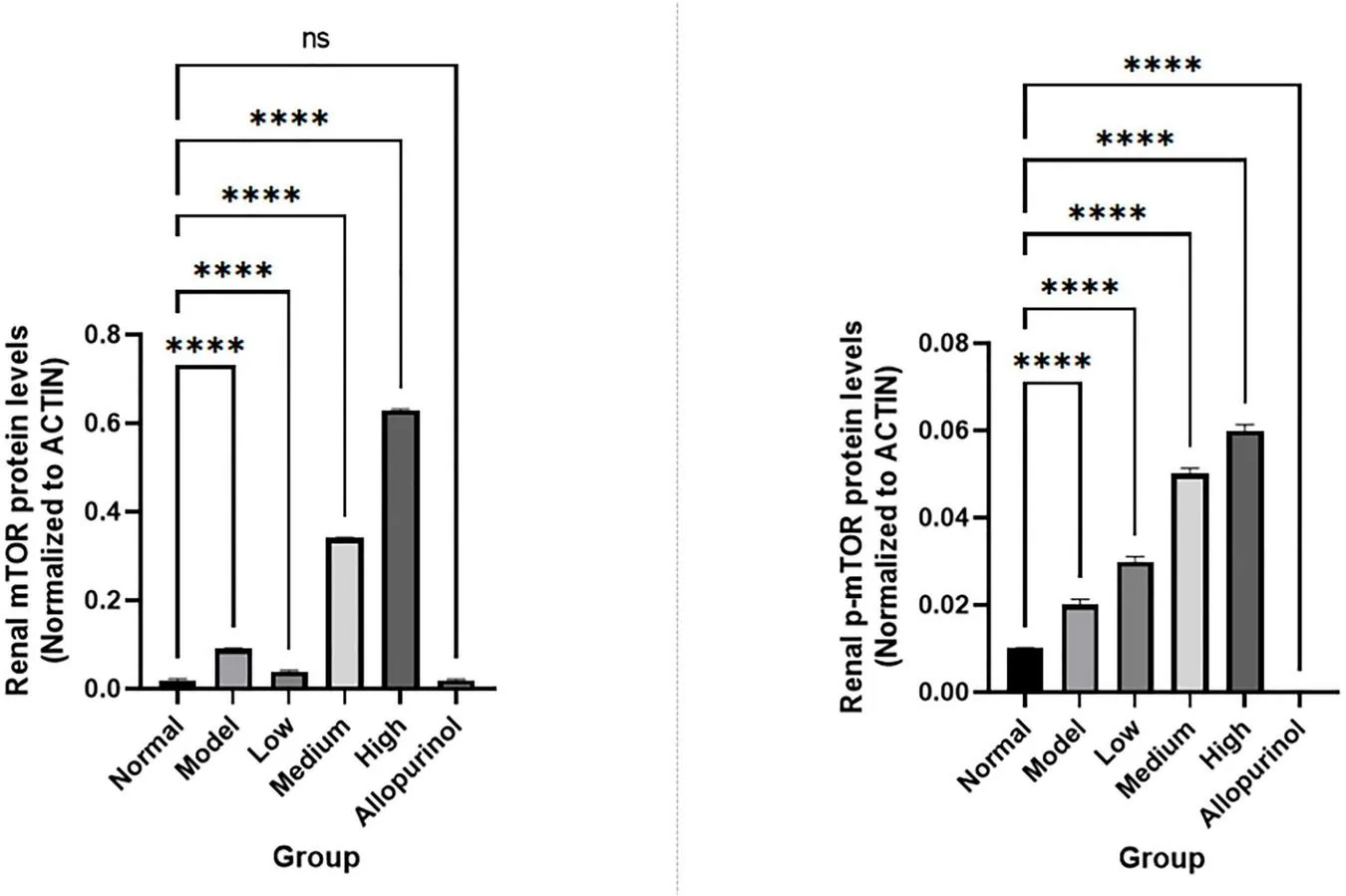
Effects of tea polyphenols on protein expression levels of mTOR, p-mTOR in HUA mice. ****Denotes a statistically significant difference at the *p* < 0.0001 level.

**FIGURE 10:**
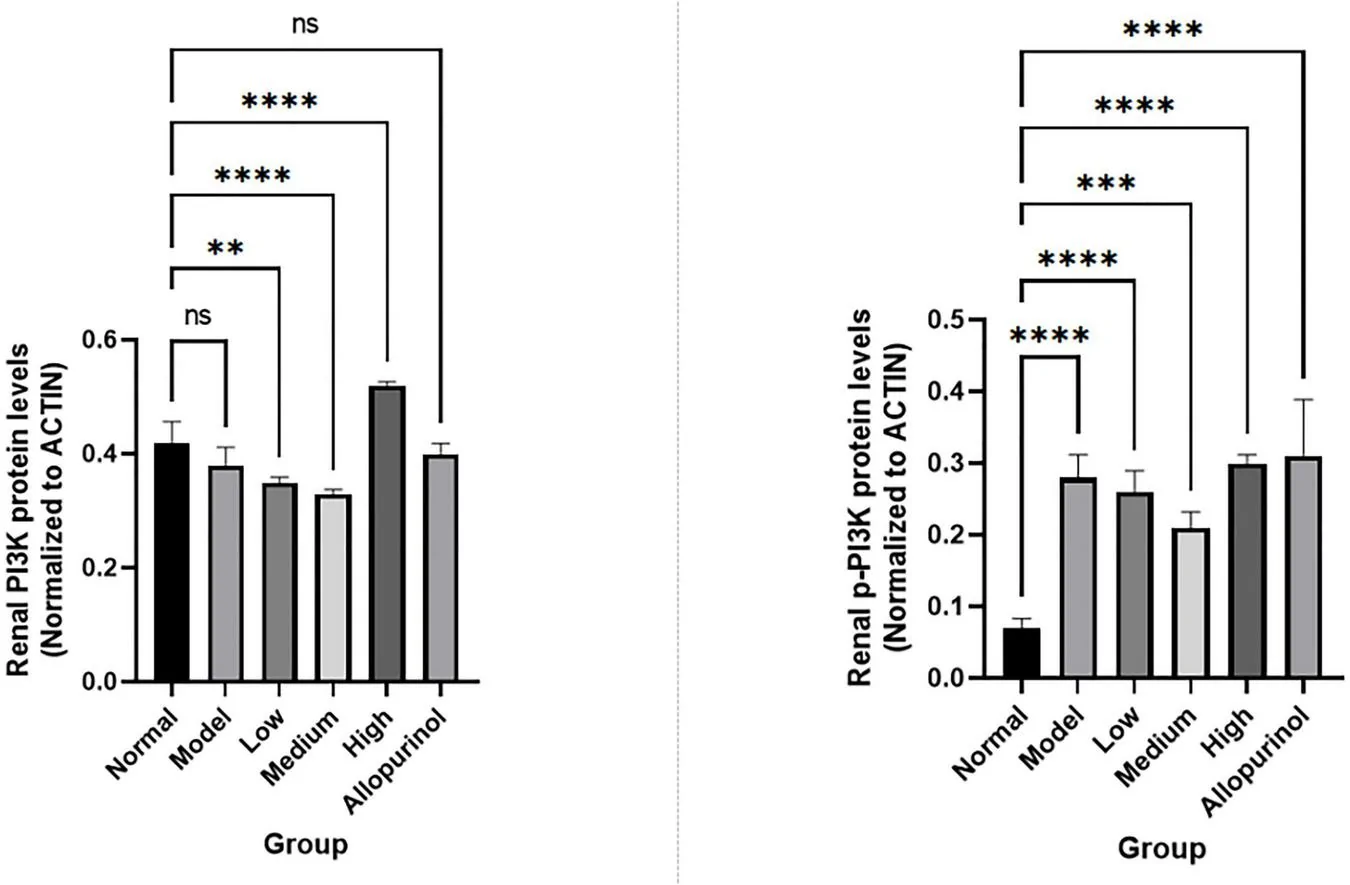
Effects of tea polyphenols on protein expression levels of PI3K, p-PI3K in HUA mice. *p < 0.05; **p < 0.01; ***p < 0.001; ****p < 0.0001.

Western blot analysis (Figure 7) showed that p-AKT/AKT and p-PI3K/PI3K ratios were significantly elevated in the model group compared with normal controls (Figures 8–10), confirming PI3K/AKT activation in HUA kidneys. Low- and medium-dose TP significantly reduced both ratios. The high-dose TP group, however, exhibited elevated p-AKT/AKT, p-PI3K/PI3K, and p-mTOR/mTOR ratios, suggesting pathway overactivation at this dose. Total AKT, PI3K, and mTOR protein levels did not differ among groups, confirming that the changes were in phosphorylation, not total protein expression.

## 4 Discussion

Currently available urate-lowering drugs, such as allopurinol, are associated with hepatorenal toxicity and hypersensitivity reactions (8), highlighting the need for safer dietary alternatives. To address this gap, we integrated network pharmacology with an *in vivo* mouse model to characterize the anti-hyperuricemic effects of oolong tea polyphenols (TP). Target prediction yielded 59 candidate anti-HUA targets, among which the PI3K/AKT/mTOR signaling axis emerged as the dominant regulatory hub. This finding is consistent with prior evidence that epigallocatechin gallate (EGCG), the principal catechin in tea, modulates renal urate transporters and exerts metabolic benefits via PI3K/AKT-dependent mechanisms (11, 20).

The *in vivo* data revealed a non-monotonic dose–response. The medium dose (1 g⋅kg^−1^⋅d^−1^) suppressed COX-2 and TNF-α expression and XOD activity most effectively, with concurrent hepatic and renal protection confirmed by histopathology. The high dose (2 g⋅kg^−1^⋅d^−1^), although it elicited the greatest reduction in serum urate, paradoxically raised IL-1β and drove overactivation of PI3K/AKT/mTOR signaling. This biphasic response—pathway inhibition at low and medium doses, activation at the high dose—is characteristic of polyphenol hormesis. At moderate concentrations, tea catechins are thought to act as antioxidants that dampen receptor-mediated PI3K/AKT signaling and limit oxidative-stress-driven pathway activation. At supraphysiological concentrations, however, catechins may undergo auto-oxidation, generating reactive oxygen species that elicit compensatory pathway activation. This pattern mirrors the pro-oxidant and hepatotoxic effects of high-dose green tea polyphenols documented in rodent studies, and is in line with the hormetic PI3K/AKT regulation reported for other natural compounds such as ginsenosides. Collectively, these data delineate a therapeutic window with a defined upper safety limit, within which the medium dose offers the optimal balance of efficacy and safety.

This study has several limitations that should be acknowledged. First, the network pharmacology pipeline, although systematic, is inherently predictive: the 59 overlapping targets were derived computationally, and none of the candidate hub genes was experimentally validated by qPCR, western blotting, or knockdown assays. The direct molecular target(s) of TP therefore remain unidentified, and the mechanistic link between TP and the PI3K/AKT/mTOR axis is correlative rather than causal. A further mismatch concerns the inputs to the computational and *in vivo* arms: target prediction relied on seven catechin monomers (EGCG, GCG, ECG, EGC, EC, C, and CAF), whereas the animal study used a commercial oolong tea polyphenol mixture for which individual monomer composition was not quantified. The ingested dose of any individual monomer therefore cannot be back-calculated from the total polyphenol dosage, which weakens the linkage between predicted targets and observed *in vivo* effects. Future work will integrate HPLC- or LC-MS-based monomer quantification to establish causal mechanisms and to permit dose-response attribution at the level of individual catechins. Second, the renal urate transport system—including URAT1 (SLC22A12), GLUT9 (SLC2A9), OAT1 (SLC22A6), and ABCG2—was not examined; inferences about uric acid excretion therefore rest on indirect indicators rather than on direct transporter measurement. Third, although widely adopted, the potassium oxonate-induced mouse model does not fully reproduce the chronic, multifactorial nature of human HUA. In addition, serum creatinine was not assayed because of an unintended omission in the experimental design, restricting renal function assessment to blood urea nitrogen and histopathology. The incomplete compositional characterization of the crude TP extract and the lack of a dedicated placebo control further limit interpretation. Addressing these gaps in subsequent work will require target deconvolution, conditional modulation of the pathway, comprehensive monomer profiling, and detailed pharmacokinetic evaluation.

Taken together, a medium dose of oolong tea polyphenols (TP) lowered serum urate and curbed inflammation in HUA mice without compromising hepatic or renal function. The reversal observed at the high dose—loss of benefit, rise in IL-1β, and overactivation of PI3K/AKT/mTOR signaling—shows that the therapeutic window is narrow, and careful dose selection will be required before clinical translation can be considered.

## 5 Outlook

Future work should build on the dose-dependent and hormetic patterns observed here. Pharmacological inhibitors and genetic knockdown experiments should be used to test whether specific nodes in the PI3K/AKT/mTOR cascade are required for the urate-lowering effect. In parallel, quantification of renal urate transporters would clarify whether TP also promotes uric acid excretion. Finally, high-throughput screening combined with *in vivo* metabolomic profiling could identify which TP metabolites are responsible for the biphasic response and help define a safe and effective dose range for clinical use.

## Data Availability

Drug–target data were obtained from the following databases: PubChem (https://pubchem.ncbi.nlm.nih.gov/), PharmMapper (https://www.lilab-ecust.cn/pharmmapper/submitfile.html/), and SwissTargetPrediction (http://www.swisstargetprediction.ch/). Disease-related gene data were acquired from DrugBank (https://go.drugbank.com/), GeneCards (https://www.genecards.org/), TTD (http://db.idrblab.net/ttd/), and PharmGKB (https://www.pharmgkb.org/).

## Footnotes

1
https://pubchem.ncbi.nlm.nih.gov/

2
^2^ https://www.lilab-ecust.cn/pharmmapper/submitfile.html/

3
http://www.swisstargetprediction.ch/

4
https://www.uniprot.org/

5
https://go.drugbank.com/

6
https://www.genecards.org/

7
http://db.idrblab.net/ttd/

8
https://www.pharmgkb.org/

9
https://www.string-db.org/
