# Correction: Network pharmacology-based prediction and experimental validation of the anti-hyperuricemic effects of oolong tea polyphenols

**DOI:** 10.3389/fnut.2026.1973561

**Published:** 2026-09-01

**Authors:** Fenqi Lin, Duoer Lu, Jia Chen, Dongming Lu, Xiangjun Liu, Shiyu Lin, Langming Wu, Leisi Sun, Yueji Luo

**Affiliations:** 1 Changsha Medical University, Changsha, Hunan, China; 2 Hunan Provincial Key Laboratory of the Traditional Chinese Medicine Agricultural Biogenomics, Changsha Medical University, Changsha, Hunan, China

**Keywords:** dose-response, hyperuricemia, network pharmacology, oolong tea polyphenols, PI3K/Akt/mTOR signaling pathway, uric acid

Affiliation Changsha Medical University, Changsha, Hunan, China was omitted from the paper. This affiliation has now been added for author(s) Fenqi Lin, Duoer Lu, Jia Chen, Dongming Lu, Xiangjun Liu, Shiyu Lin, Langming Wu, Leisi Sun and Yueji Luo.

Affiliation Hunan Provincial Key Laboratory of the Traditional Chinese Medicine Agricultural Biogenomics, Changsha Medical University, Changsha, Hunan, China was omitted from the paper. This affiliation has now been added for author(s) Fenqi Lin and Yueji Luo.

Affiliation College of Traditional Chinese Medicine, Changsha Medical University, Changsha, Hunan, China was erroneously included in the paper. This affiliation has now been removed for author(s) Fenqi Lin, Duoer Lu, Jia Chen, Dongming Lu, Xiangjun Liu, Shiyu Lin, Langming Wu, Leisi Sun.

Affiliation Basic Medical School, Changsha Medical University, Changsha, Hunan, China was erroneously included in the paper. This affiliation has now been removed for author(s) Yueji Luo.

The original version of this article has been updated.

